# Network pharmacology reveals that Berberine may function against Alzheimer’s disease via the AKT signaling pathway

**DOI:** 10.3389/fnins.2023.1059496

**Published:** 2023-05-04

**Authors:** Wei Wei, Jiu-xiu Yao, Ting-ting Zhang, Jia-yu Wen, Zhen Zhang, Yi-miao Luo, Yu Cao, Hao Li

**Affiliations:** ^1^Wangjing Hospital, China Academy of Chinese Medical Science, Beijing, China; ^2^Institute of Geriatrics, Xiyuan Hospital, China Academy of Chinese Medical Science, Beijing, China; ^3^College of First Clinical Medicine, Shandong University of Traditional Chinese Medicine, Jinan, Shandong, China

**Keywords:** Berberine, Alzheimer’s disease, AKT, pharmacology, neuroprotective effect

## Abstract

**Objective:**

To investigate the mechanism underlying the effects of berberine (BBR) in the treatment of Alzheimer’s disease (AD).

**Methods:**

3 × Tg AD mice were treated with BBR for 3 months, then the open field test (OFT), the novel object recognition test (NOR) and the Morris water maze (MWM) test were performed to assess behavioral performance. Hematoxylin–eosin (HE) staining, Nissl staining were used to examine histopathological changes. The pharmacological and molecular properties of BBR were obtained from the TCMSP database. BBR-associated AD targets were identified using the PharmMapper (PM), the comparative toxicogenomics database (CTD), DisGeNet and the human gene database (GeneCards). Core networks and BBR targets for the treatment of AD were identified using PPI network and functional enrichment analyses. AutoDock software was used to model the interaction between BBR and potential targets. Finally, RT-qPCR, western blotting were used to validate the expression of core targets.

**Results:**

Behavioral experiments, HE staining and Nissl staining have shown that BBR can improve memory task performance and neuronal damage in the hippocampus of AD mice. 117 BBR-associated targets for the treatment of AD were identified, and 43 genes were used for downstream functional enrichment analysis in combination with the results of protein–protein interaction (PPI) network analysis. 2,230 biological processes (BP) terms, 67 cell components (CC) terms, 243 molecular function (MF) terms and 118 KEGG terms were identified. *ALB*, *EGFR*, *CASP3* and five targets in the PI3K-AKT signaling pathway including *AKT1*, *HSP90AA1*, *SRC*, *HRAS*, *IGF1* were selected by PPI network analysis, validated by molecular docking analysis and RT-q PCR as core targets for further analysis. *Akt1* mRNA expression levels were significantly decreased in AD mice and significantly increased after BBR treatment (*p* < 0.05). Besides, AKT and ERK phosphorylation decreased in the model group, and BBR significantly increased their phosphorylation levels.

**Conclusion:**

*AKT1*, *HSP90AA1*, *SRC*, *HRAS*, *IGF1* and *ALB*, *EGFR*, *CASP3* were core targets of BBR in the treatment of AD. BBR may exert a neuroprotective effect by modulating the ERK and AKT signaling pathways.

## Introduction

Alzheimer’s disease International (ADI) estimates that approximately 50 million people worldwide have dementia and that this number will triple by 2050, placing a great burden on families and society. Despite the urgent need, the development of new drugs faces significant challenges. Currently, only cholinesterase inhibitors and N-methyl-D-aspartate receptor antagonists have been approved for cognitive enhancement, although tremendous progress has been made in the understanding of the molecular mechanisms (Scheltens et al., 2021). In addition to the classical targets related to Aβ and tau protein hyperphosphorylation, lysosomal pathways, autophagy, apoptosis, transcription factor EB (TFEB), and TREM2 are also potential therapeutic pathways and targets for Alzheimer’s disease (AD) (Singh et al., 2019a; Rai et al., 2021). Multi-target effect of Traditional Chinese medicine (TCM) has been attributed with unique benefits in the treatment of AD. Pharmacological data show that various formulas, extracts and compounds can alleviate symptoms and improve the quality of life of AD patients (Alexiou et al., 2019; Pei et al., 2020).

Recently, Berberine (BBR) has attracted much interest due to its pharmacological effects in the treatment and/or management of AD (Singh et al., 2019b, 2021a). BBR is an isoquinoline alkaloid and the main active component of several well-known herbs used in TCM, such as *Coptis chinensis* Franch (Huanglian) and *Phellodendron sinii* Y.C. Wu (Huangbo). Previous data suggest that BBR is a promising therapeutic agent for the treatment of bacterial diarrhea, cardiovascular and metabolic diseases (Jiang et al., 2011; Feng et al., 2019; Xu et al., 2021). BBR has therapeutic potential in the treatment of AD by targeting amyloid beta plaques, neurofibrillary tangles, neuroinflammation, and oxidative stress (Zhu and Qian, 2006; Jiang et al., 2015). However, the molecular basis of these effects has not been elucidated.

**Figure 1:**
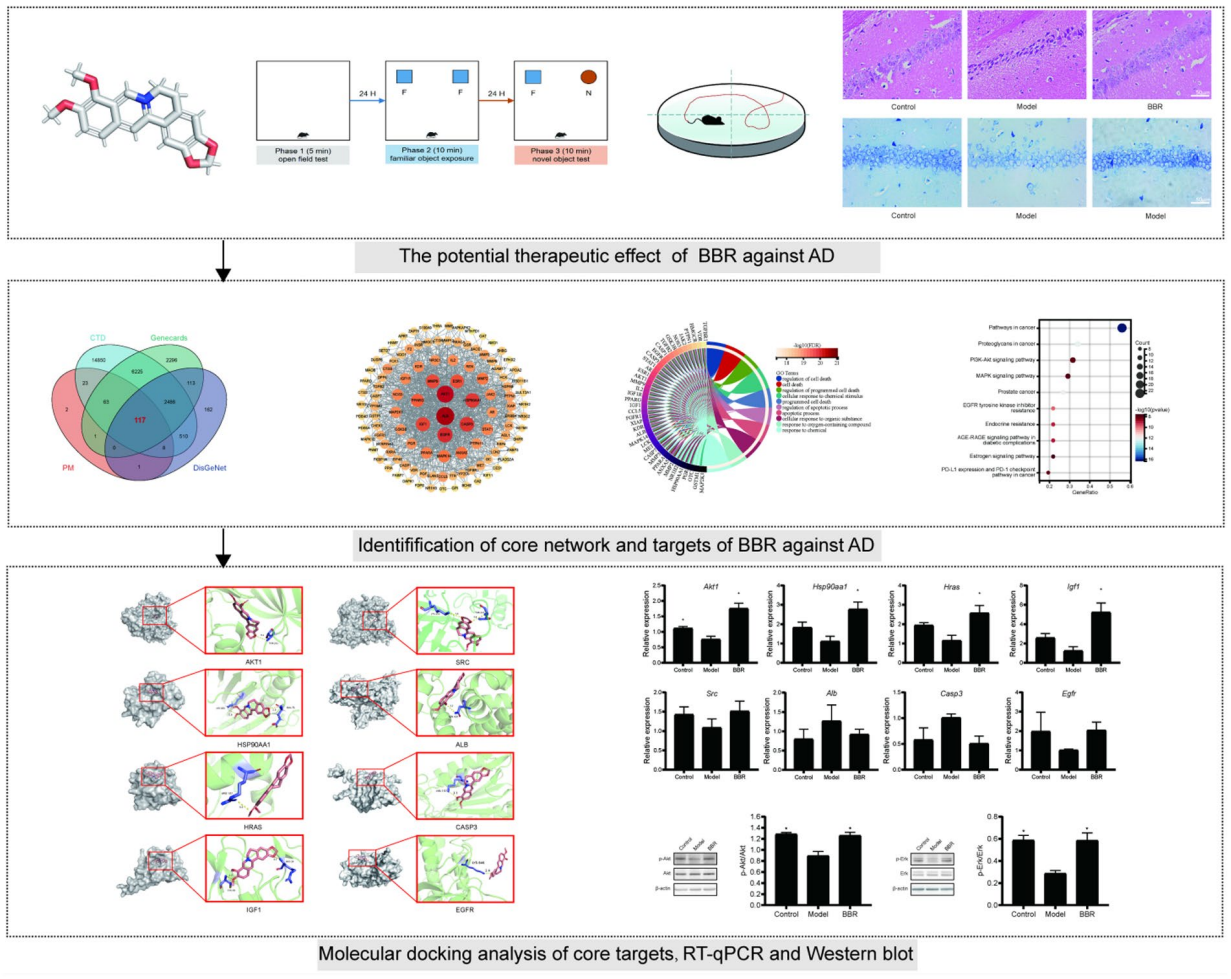
Workflow of the study.

To further explore the therapeutic potential and efficacy basis of BBR in AD, we used network pharmacology to investigate the mechanisms underlying the multiple effects of BBR in this work. Network pharmacology is an emerging field of pharmacology that can be used to study drug action and interaction with targets (Berger and Iyengar, 2009). The dose of BBR used in our experiments was based on a previous research report (Kong et al., 2004). The workflow of this study is presented in Figure 1. This study may provide the basis for the development of BBR applications in the prevention and treatment of AD.

## Materials and methods

### Animals

Twenty 11–12 months 3 × Tg AD female mice (No: 14002A) were randomly divided in 2 groups: the 3 × Tg AD model group and the BBR group (50 mg∙kg^−1^∙d^−1^). Ten age-matched wild-type female C57 mice were used as controls. All animals were purchased from Beijing HFK Bioscience Co., Ltd. All animals were caged in SPF barrier-protected facilities with a temperature of (22 ± 3) °C, relative humidity of (50 ± 10) % and automatic light cycles (12 h light/dark). Food and water were freely available. This experiment started after 1 week of adaptive feeding. All experiments were carried out according to animal care guidelines and were approved by the Ethics Committee of the Xiyuan Hospital of the China Academy of Chinese Medical Sciences (No. 2022XLC020-2). Each mouse was fed 0.1 ml/10 g body weight by gavage for 3 months, and the BBR group received the corresponding drug, while the 3 × Tg AD model group and the control group received equal amounts of distilled water. The final numbers of animals in the control group, model group and BBR group were 9, 9 and 7, respectively due to mishandling.

### Chemical reagents

Berberine at ≥98% purity (LDSW220209-1) was supplied by Shaanxi Langde Biotechnology Co., Ltd., and the HE staining kit was purchased from Servicebio (G1003).

### Behavioral experiments

The open field test (OFT) was performed by placing the mice in a box (40 cm L × 40 cm W × 40 cm H). The test area in the box was divided into 16 squares (10 × 10 cm) by the computer software and the four innermost squares were defined as the central area. During 5 min OFT test, total distance (cm), total movement (s), speed (cm / s), distance in the center (cm), and time in the center (s) were calculated.

Twenty-four hours after OFT test, a novel object recognition test (NOR) was performed. This test consisted of an exposure phase to a familiar object, followed by a phase of exposure to a novel object the next day. Each phase lasted 10 min. During the exposure phase to a familiar object, mice were placed in the same box with two identical objects in two parallel corners. 24 h later, one of the two objects was replaced by a novel one. The preference index (PI) and the recognition index (RI) were recorded. PI was defined as the time spent exploring two objects during the exposure phase to a familiar object, while RI was calculated as follows: *T_novel_/(T_novel_ + T_familiar_)*. Notes: *T_novel_* and *T_familiar_* indicate the time spent with the novel and familiar objects during the exposure phase to the novel object.

Next, the Morris water maze (MWM) test was used to assess spatial memory of mice. The facility consists of a white tank (diameter 120 cm, height 50 cm), an automatic camera, and a computer analysis system. The pool was divided into four quadrants with specific markers, respectively, and a cylindrical escape platform (diameter 10 cm, height 15 cm) was placed in the first quadrant 1 cm beneath the water filled with nontoxic white dye. For training, spatial navigation experiments were performed for five consecutive days; each mouse was trained four trials per day for 90 s each using different entry points. The mice were placed in the water facing the wall of the pool, and the time between entering the water and finding the escape platform (with all limbs on the platform for 6 s; escape latency) was recorded using a video tracking system. On day 6, the escape platform was removed for spatial exploration experiments; mice were placed in the water facing the wall of the pool (at the midpoint of the third quadrant), and the number of times the mouse crossed the original platform area within 90 s was recorded, the time in the target quadrant and distance moved in the target quadrant were also recorded to examine memory of the target quadrant where the original platform was located. A quiet environment with a stable light source and a water temperature of 23 ± 1°C were maintained during experiments.

### Tissue preparation

After the behavioral experiments, the brains were quickly removed. For each group, half of the brains were fixed in 4% paraformaldehyde, and the other half were snap frozen in liquid nitrogen and stored at −80° C for further analysis.

### Histomorphological observation of brain tissue

Brain tissues were embedded in paraffin and sectioned. Then sections were processed as follows: Xylene for 20 min, two times; 100% ethanol for 5 min, two times; 75% ethanol for 5 min; and tap water rinsing. Sections were subjected to hematoxylin–eosin (HE) and toluidine blue (Nissl) staining. Hematoxylin–Eosin Staining Kit (G1003; Wuhan Servicebio Technology Co., Ltd., Wuhan, China) was used for HE staining, and Toluidine blue staining solution (G1032; Wuhan Servicebio Technology Co., Ltd.) was used for Nissl staining. Sections were finally sealed with neutral gum and examined under an upright optical microscope (NIKON ECLIPSE E100) for image acquisition.

### Data on the pharmacological and molecular properties of BBR

MOL001454 (CAS, 2086-83-1) was found by searching for the chemical name “berberine” in the traditional Chinese medicine systems pharmacology database (TCMSP),^1^ and absorption, distribution, metabolism, and excretion (ADME) parameters for BBR were also obtained. The molecular structure of BBR was downloaded from the PubChem database^2^ and dealed with PyMOL2.4.0 software.

### Identification and collection of potential targets of BBR

Potential BBR targets were identified and collected using the PharmMapper platform,^3^ which is an integrated pharmacophore matching platform including targets extracted from TargetBank, DrugBank, BindingDB, and PDTD database and over 7,000 receptor-based pharmacophore models (Liu et al., 2010; Wang et al., 2017). All predicted targets were imported in EXCEL to establish the BBR target database.

### BBR-associated targets of Alzheimer’s disease

The comparative toxicogenomics database (CTD),^4^ DisGeNet^5^ (Piñero et al., 2015, 2017, 2020, 2021) and the human gene database (GeneCards)^6^ were used to identify AD targets with the keyword ‘Alzheimer’s disease’. Then, BBR-associated targets of AD were generated by intersecting BBR targets with the above targets of Alzheimer’s disease.

### Network construction and analysis of the protein–protein interaction

BBR-associated targets of AD were imported into STRING,^7^ and protein–protein interaction (PPI) data were exported under the condition that the minimum required interaction score was 0.700. We then built a network diagram using the Cytoscape 3.7.2 software. In addition, the MCODE and cytoHubba plug-ins were used to screen important PPI network modules.

### Functional enrichment analysis

The biological process (BP), molecular function (MF), and cellular component (CC) are three important parts of gene ontology (GO). GO and Kyoto Encyclopedia of Genes and Genomes (KEGG) pathway analysis were used for functional enrichment analysis. The R 3.6.3 software was used for statistical analysis and visualization; the ggplot2 package 3.3.3 and the clusterProfiler package 3.14 were used. Significant terms were identified under the condition that false discovery rate (FDR) was <0.05. The top terms identified were visualized in a diagram.

### Molecular docking verification

The molecular structure of BBR was downloaded from TCMSP, while the crystal structures of target proteins were obtained from the Protein Data Bank (PDB) database.^8^ PyMOL 2.4.0 software,^9^ Auto Dock Tools 1.5.7 software were used for pre-docking molecular processing to obtain PDBQT file. The Auto Dock Vina software 1.1.2^10^ was then used for molecular docking and calculation of the affinity score. A lower affinity score indicates stronger binding. The results of molecular docking were visualized by PyMOL2.4.0 software. We calculated the RMSD (Root Mean Square Deviation) using PyMOL2.4.0 to verify the reliability, and RMSD <2A was considered reliable.

### RT-qPCR analysis

An RNA extraction kit (Servicebio, G3640-50 T) was used to extract total RNA from brain tissue. ReverTra Ace qPCR RT Master Mix (TOYOBO, FSQ-201) was used for reverse transcription to generate cDNA templates and then real-time PCR was performed under the following conditions: 95° C for 10 min, followed by 40 cycles of 95 ° C for 15 s and 60 ° C for 60 s. Real-time PCR was performed using Applied Biosystem 7,500 Real-Time PCR System, and the relative expression of mRNA was calculated using the 2^−ΔΔCt^ method. The primer sequences of all target genes are shown in Table 1.

**Table 1:** Primer sequences of all target genes.

Gene	Forward Primer (5′-3′)	Reverse Primer (5′-3′)
*Hsp90*	TTTACTCTGCCTATTTGGTTGCTG	CACAAAGAGAGTAATGGGATAGCC
*Akt1*	TTTGGGAAGGTGATTCTGGTG	CAGGACACGGTTCTCAGTAAGC
*Src*	AGATCACTAGACGGGAATCAGAGC	GCACCTTTTGTGGTCTCACTCTC
*Hras*	AGTACAGGGAGCAGATCAAGCG	TGGCTGATGTTTCAATGTAGGG
*Igf1*	GACCGCACCTGCAATAAAGATAC	CCTGTGGGCTTGTTGAAGTAAA
*Alb*	AACAAGAGCCCGAAAGAAACG	CTGGCAACTTCATGCAAATAGTG
*Casp3*	TGGAATGTCATCTCGCTCTGGT	GAAGAGTTTCGGCTTTCCAGTC
*Egfr*	CCGAAACTACGTGGTGACAGAT	TGCCATTACAAACTTTGCGAC
*Gapdh*	CCTCGTCCCGTAGACAAAATG	TGAGGTCAATGAAGGGGTCGT

### Western blotting

Brain tissues were lysed on ice in lysis buffer and then centrifuged at 12,000 rpm for 20 min at 4°C. The protein content was determined using the bicinchoninic acid (BCA) method (G2026, Servicebio, Wuhan, China). The extracted proteins were subjected to SDS-PAGE electrophoresis, transferred to 0.45 μm polyvinylidene difluoride membranes (Millipore, Bedford, MA), and then blocked with 5% BSA in TBST for 60 min. The membranes were subsequently incubated with primary antibodie. The primary antibodies were listed as follows: Erk 1/2 (diluted 1:2000, rabbit, Cell Signaling Technology, Cat# 4695), p-Erk 1/2 (diluted 1:2000, rabbit, Cell Signaling Technology, Cat# 4370), Akt (diluted 1:5000, rabbit, proteintech, Cat No. 10176-2-AP), p-Akt (diluted 1:5000, Mouse, proteintech, Cat No: 66444-1-Ig), or β–actin (diluted 1:10000, Mouse, proteintech, Cat No: 66009-1-Ig) overnight at 4°C. The membrane was incubated with the HRP conjugated secondary antibodies, goat anti-mouse (diluted 1:10000, Abbkine, China) or goat anti-rabbit IgG (diluted 1:10000, Abbkine, China) for 90 min after the membrane had been washed with TBST 4 times for 10 min each. The blots of proteins of interest were visualized using sensitive ECL western HRP substrate (# 17046, ZENBIO, Chengdu, China). Quantitative analysis of protein bands were performed using ImageJ software.

### Statistical analysis

Data were statistically analyzed using SPSS 22.0 software (IBM Corp., Armonk, NY, USA). Quantitative data were expressed as mean with standard error of mean (SEM). Comparison between two groups were performed using *t* test. The MWM latency data were analyzed using a repeated-measures analysis of variance. All tests were two-sided with an α of 0.05 and statistical significance threshold of *p* < 0.05.

## Results

### Evaluation of druggability of BBR

**Figure 2:**
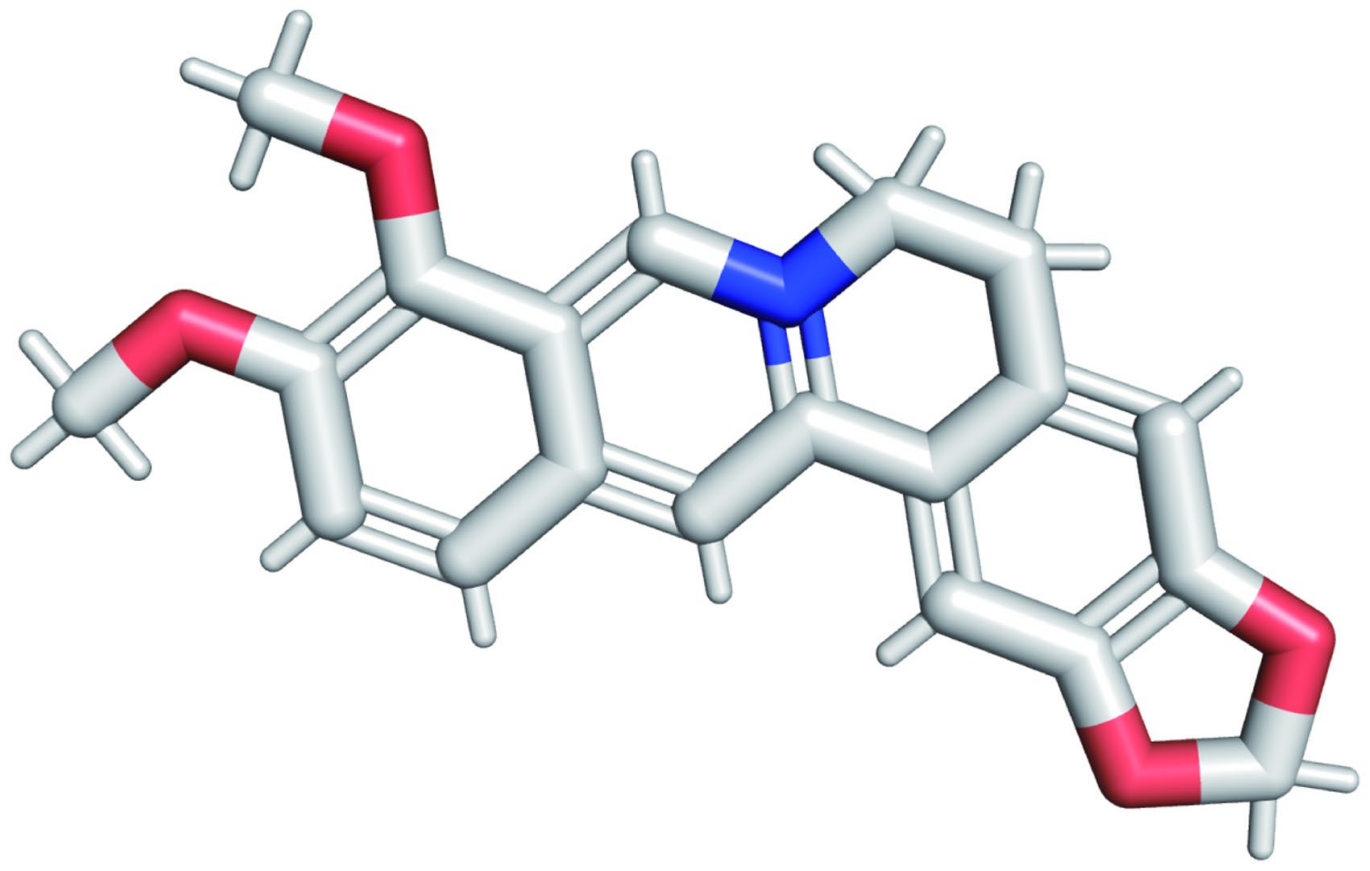
The molecular structure of BBR. The red structure in the figure represents the oxygen atom and the blue structure represents the hydrogen atom.

The pharmacological and molecular properties of BBR were obtained from TCMSP (Ru et al., 2014) and are as follows: molecular weight (MW) = 336.39, oral bioavailability (OB) = 36.86%, drug-likeness (DL) = 0.78, blood brain barrier (BBB) = 0.57, half-life (HL) = 6.57. BBR half-life (t_1/2_) is in the mid-elimination group. The molecular structure of BBR (C_20_H_18_NO_4_) is shown in Figure 2. BBR is believed to be more druggable in the 180–500 Dalton MW range. It is a promising therapeutic agent that can act in the central nervous system and is characterized by high OR (≥20%) (Xu et al., 2012), high DL (≥0.18) (Tao et al., 2013), and strong penetration (BBB > 0.3)(Tattersall et al., 1975).

### BBR improves the performance of memory and recognition tasks in AD mice

**Figure 3:**
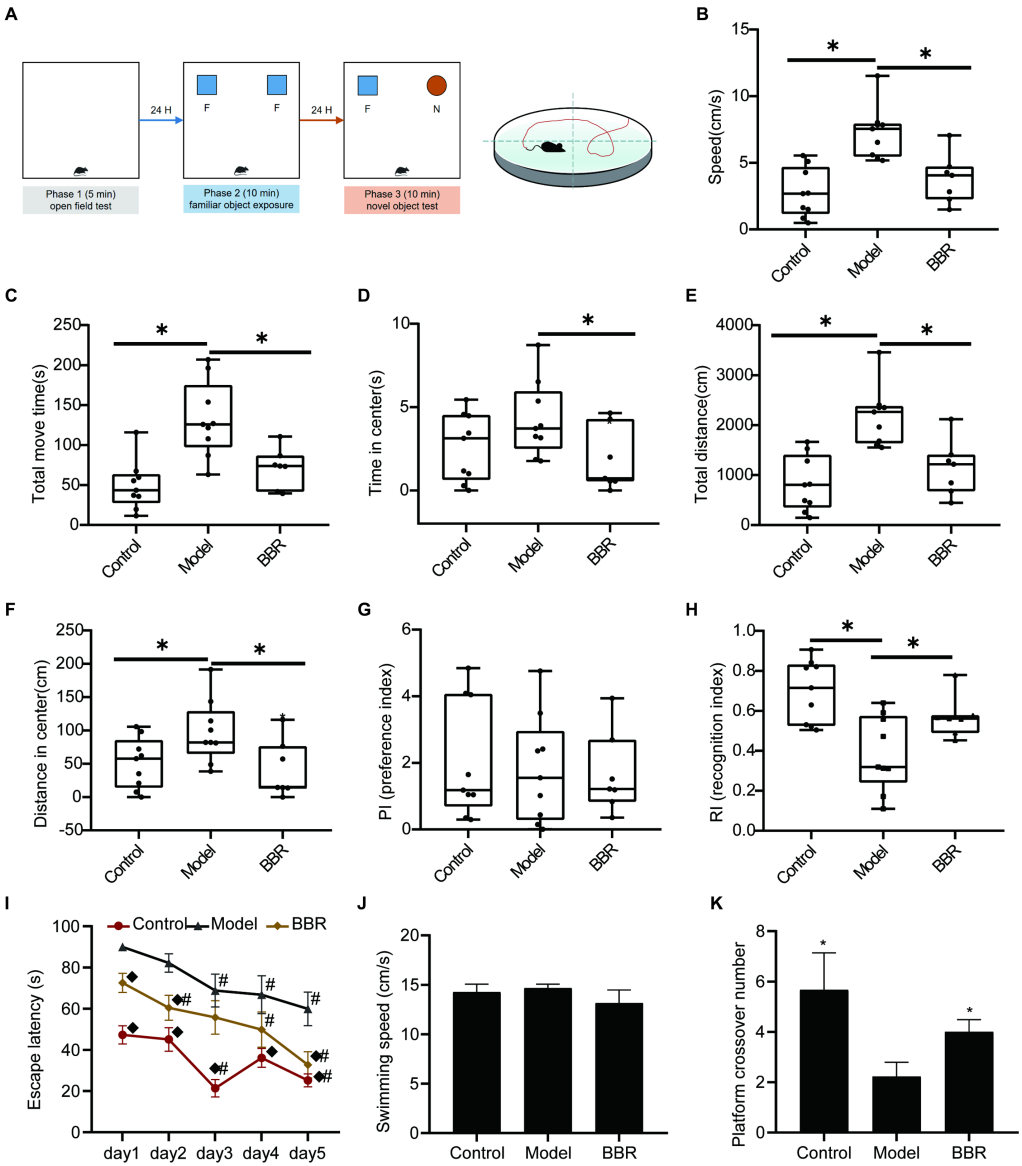
BBR improves recognition and memory task performance in AD mice. **(A)** Workflow of behavioral experiments; **(B)** Speed (cm/s), **(C)** Total move time (S), **(D)** Time in center (s), **(E)** Total distance (CM), and **(F)** Distance in center (cm) during OFT test; PI **(G)** and RI **(H)** of NOR test; Escape latency **(I)**; Swimming speed **(J)** and Platform crossover number **(K)** during the MWM test, *n* = 7–9, ^*^p < 0.05, compared with model group; ^◆^*p* < 0.05 for latency data between groups on the same day compared with the model group; ^#^*p* < 0.05 for latency data within groups compared with the first day.

The sequence of behavioral experiments is shown in Figure 3A. During the OFT test, the BBR group differed significantly from the model group in speed (*t* = 3.59, *p* = 0.003), total move time (*t* = 3.07, *p* = 0.008), time in the center (*t* = 2.27, *p* = 0.039), total distance (*t* = 3.59, *p* = 0.003), and distance in the center (*t* = 2.46, *p* = 0.0273), indicating differences in overall activity or anxiety-like behavior (Figures 3B–F). During the novel object recognition test, neither the control group nor the BBR group differed significantly from the model group in the preference index (PI) (*p* > 0.05, Figure 3G). After 3 months of BBR administration, mice in the BBR group spent significantly more time with the novel object in the NOR test as indicated by a significantly higher recognition index (*t* = −2.47, *p* = 0.028) in the NOR test (Figure 3H).

The MWM test consists of a navigation test and an exploratory experiment, which can be used to evaluate the learning memory ability of mice. In the navigation test, as shown in Figure 3I, the escape latency of all mice gradually decreased with the increasing number of training sessions, indicating that their ability to locate the platform was enhanced. The escape latency of mice in the BBR group was diminished compared to the model group and exhibited a significant difference on day 1 (*p* = 0.003), day 2 (*p* = 0.011) and day 5 (*p* = 0.007). There were no significant differences in the speed of swimming among the groups (*p* > 0.05, Figure 3J), and effects on locomotor ability could be excluded. In addition, compared to the model group, mice in the BBR group had a significantly higher number of platform crossings in the exploratory experiment on day 6 (*p* = 0.039, Figure 3K).

These results suggest that BBR alleviated the tense, manic, and anxious behaviors of AD mice and improves recognition and memory performance, although more studies are necessary to clarify the mechanisms involved. See details in Supplementary data S1.

### BBR ameliorates neuronal damage in the hippocampus

**Figure 4:**
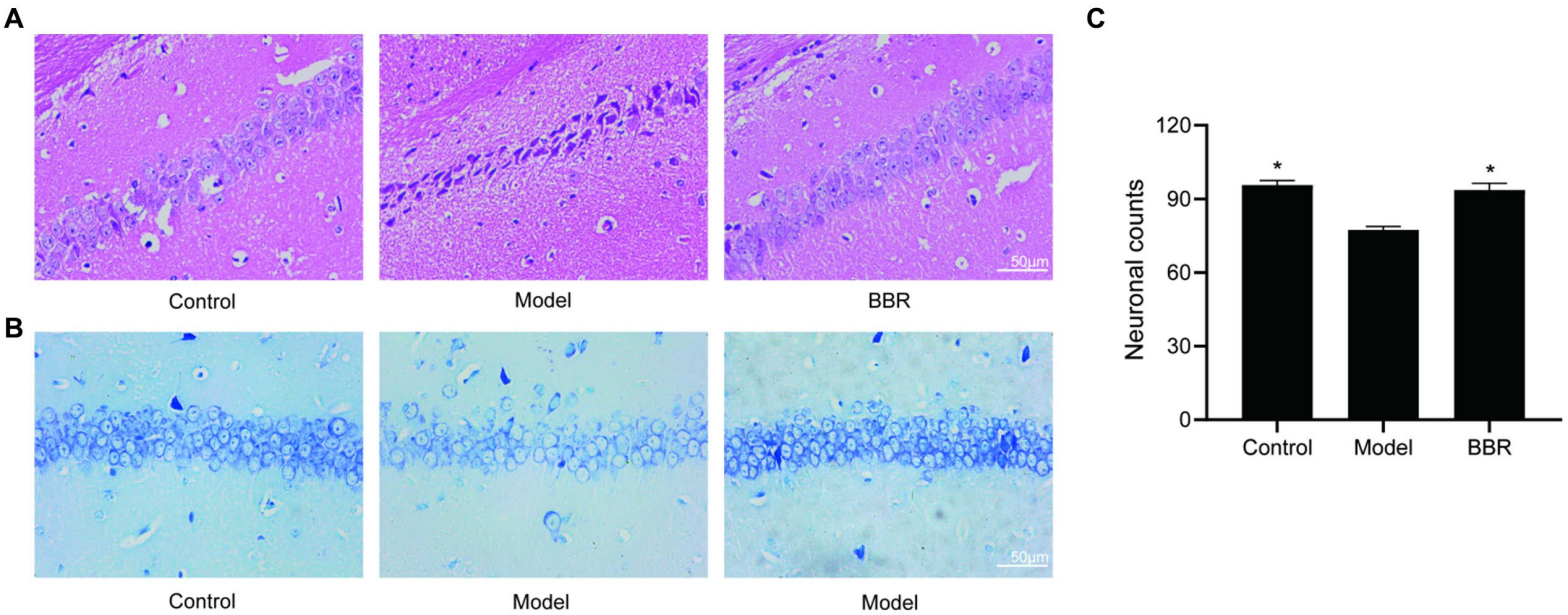
BBR ameliorated neuronal damage in the hippocampus. **(A)** HE staining and Nissl staining **(B)** and its quantitative analysis **(C)** of the CA1 region. Data were analyzed by *t* test between two groups, presented as mean ± SEM, *n* = 3, *p < 0.05 compared to the model group.

The size, density and arrangement of neurons in the hippocampus may indicate neuronal damage. Using HE staining, chromatin in the nucleus and ribosomes in the cytoplasm were stained blue-violet, while components in the cytoplasm and extracellular matrix were stained red, and the degree of staining may reflect the functional properties of hippocampal neurons. Under normal conditions, the cytoplasm is only slightly stained, but it becomes overstained when cellular senescence or neurodegenerative alterations occur. The hippocampal neurons of control mice were dense and neatly organized, with full nuclei and clear boundaries, whereas the neurons of model mice were overstained and loosely arranged, with cytoplasmic deformation and swelling. In the model mice, BBR treatment was able to reverse this phenotype and the neuronal morphology resembled that of controls (Figure 4A).

Neurons in the model group showed relatively lighter blue staining, with a reduced number of Nissl bodies. Besides, in the BBR groups, there was stronger cytoplasmic staining and neurons were densely arranged (Figure 4B). Quantitatively, in the CA1 region of the hippocampus, neuron numbers were significantly higher in the BBR (*t* = −4.07, *p* = 0.015) group compared to the model group (Figure 4C), suggesting that BBR can protect hippocampal neurons to some extent and prevent degenerative necrosis.

### Identification of BBR-associated targets of Alzheimer’s disease

**Figure 5:**
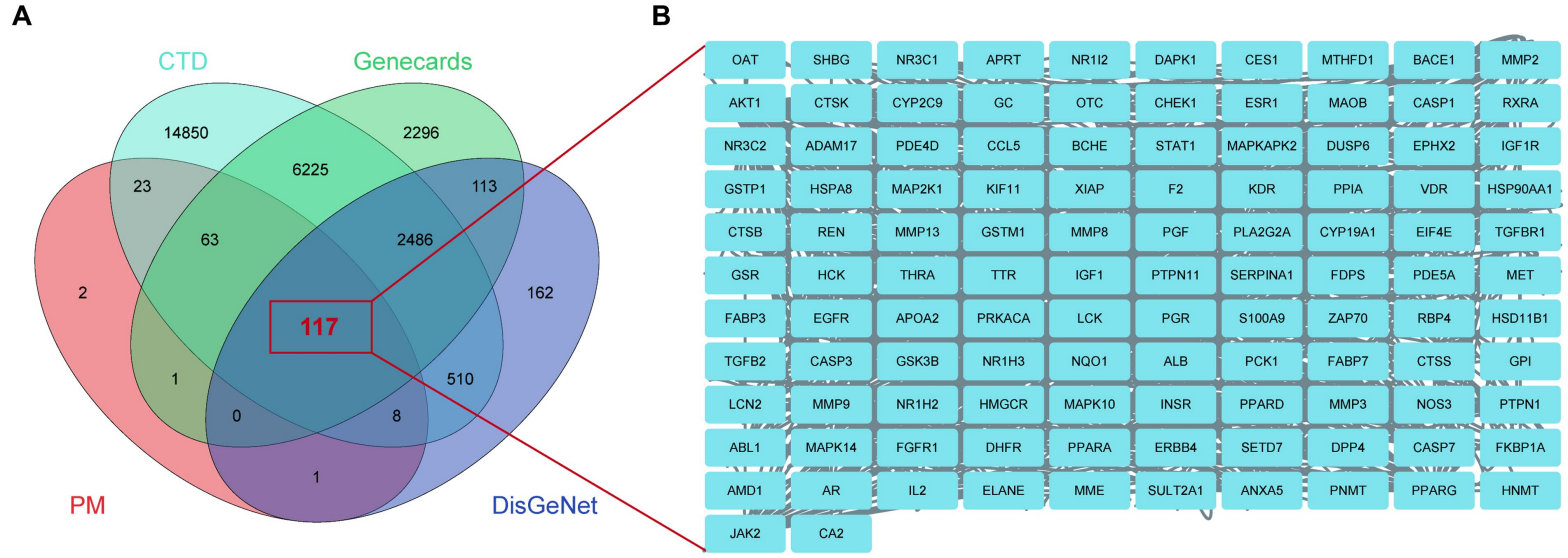
BBR-associated targets of Alzheimer’s disease. **(A)** BBR-associated targets of AD from the intersection between BBR-targets and targets of AD, zoom in to show the specific target in **(B)**.

Using the keyword ‘Alzheimer’s disease’, 215 potential BBR targets were obtained from the PM database, 24,282 AD targets from the CTD database, 3,397 AD targets from the DisGeNet database and 11,301 AD targets from GeneCards. 117 BBR-associated AD targets were generated from the intersection of these three databases using a Venn plot (Figure 5A). The names of these targets are shown in Figure 5B. See detailed gene information in Supplementary data S2.

### Identification of the core networks and targets of BBR in AD pathology

**Figure 6:**
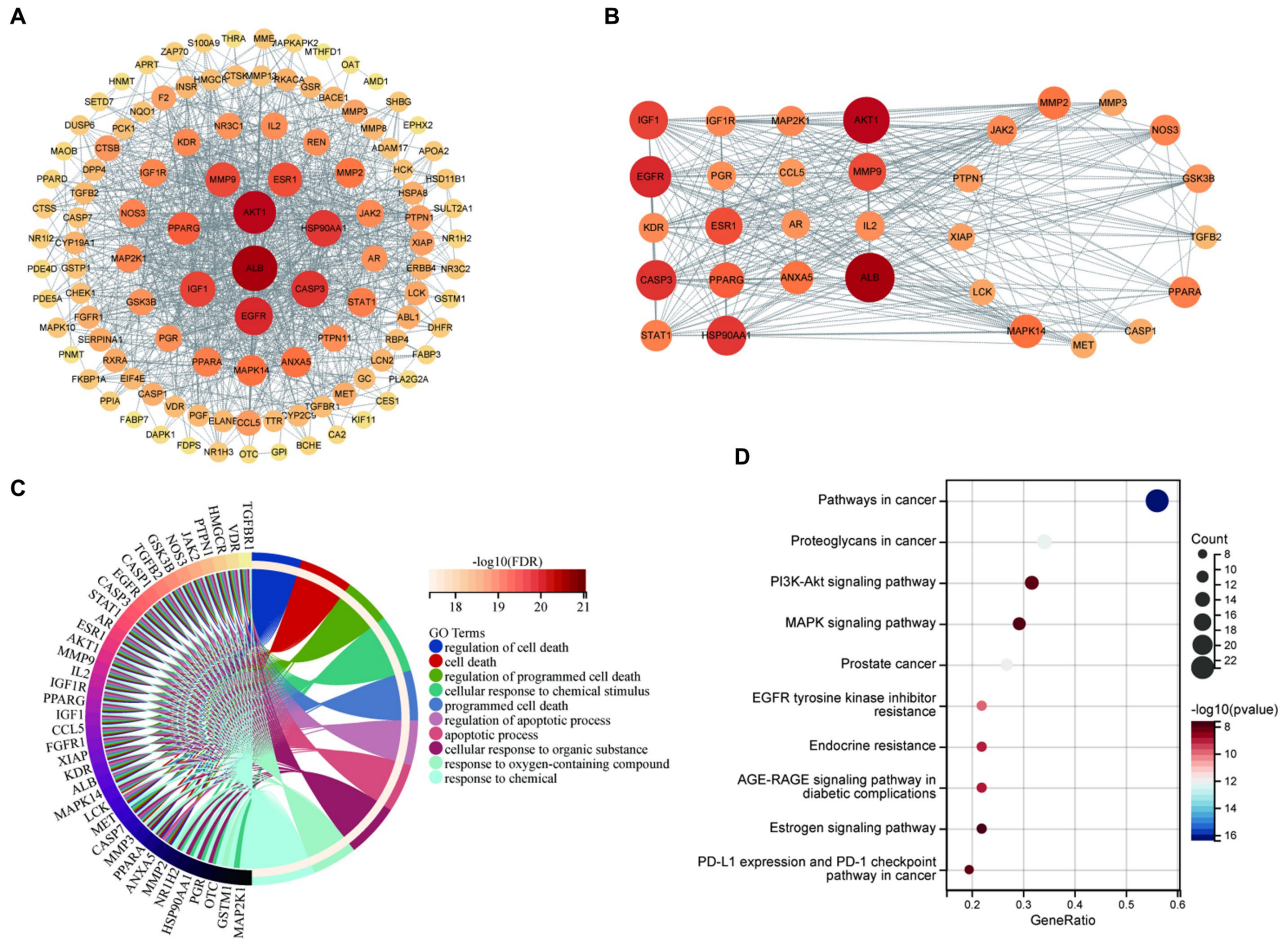
Core BBR targets in AD and GO/KEGG analysis. **(A)** PPI network of 117 BBR-associated targets of AD; **(B)** representative clusters extracted using MCODE; **(C)** GO analysis of all the targets identified above; **(D)** KEGG analysis of all the targets identified above.

Proteins interact with each other to participate in various aspects of life processes such as biological signaling, regulation of gene expression, energy and material metabolism, and cell cycle regulation. By detecting the interrelationship between target proteins, the possible pathways of action of drugs can be predicted and provide directions for in-depth research. In this study, PPI analysis between BBR-associated AD targets was performed to explore underlying mechanisms using STRING and the results are shown in Supplementary data S3. The PPI network is visualized in Figure 6A. The results of MCODE (Figure 6B) and cytoHubba are shown in Supplementary data S4. Combining the data obtained from the above algorithms, we obtained 43 genes for downstream functional enrichment analysis. In total, 2,230 BP terms, 67 CC terms, 243 MF terms (*p* < 0.01 and FDR<0.01) and 118 KEGG terms (*p* < 0.05 and FDR<0.01) (Supplementary data S5) were obtained. The top terms for each category are shown in Figure 6C: regulation of cell death, cell death, regulation of programmed cell death, cellular response to chemical stimulus, programmed cell death, regulation of apoptotic process, apoptotic process, cellular response to organic substance, response to oxygen-containing compound, response to chemical in BP enrichment analysis Furthermore, Pathways in cancer, Proteoglycans in cancer, PI3K-AKT signaling pathway, MAPK signaling pathway, Prostate cancer, EGFR tyrosine kinase inhibitor resistance, Endocrine resistance, AGE-RAGE signaling pathway in diabetic complications, Estrogen signaling pathway, PD-L1 expression and PD-1 checkpoint pathway in cancer involved in BBR treatment for AD based on KEGG pathway analysis (Figure 6D). Importantly, *ALB*, *EGFR*, *CASP3* and five members of the PI3K-Akt signaling pathway including *AKT1*, *HSP90AA1*, *SRC*, *HRA*, and *IGF1* were identified as core BBR targets in AD for further study.

### Molecular docking analysis of core targets

**Figure 7:**
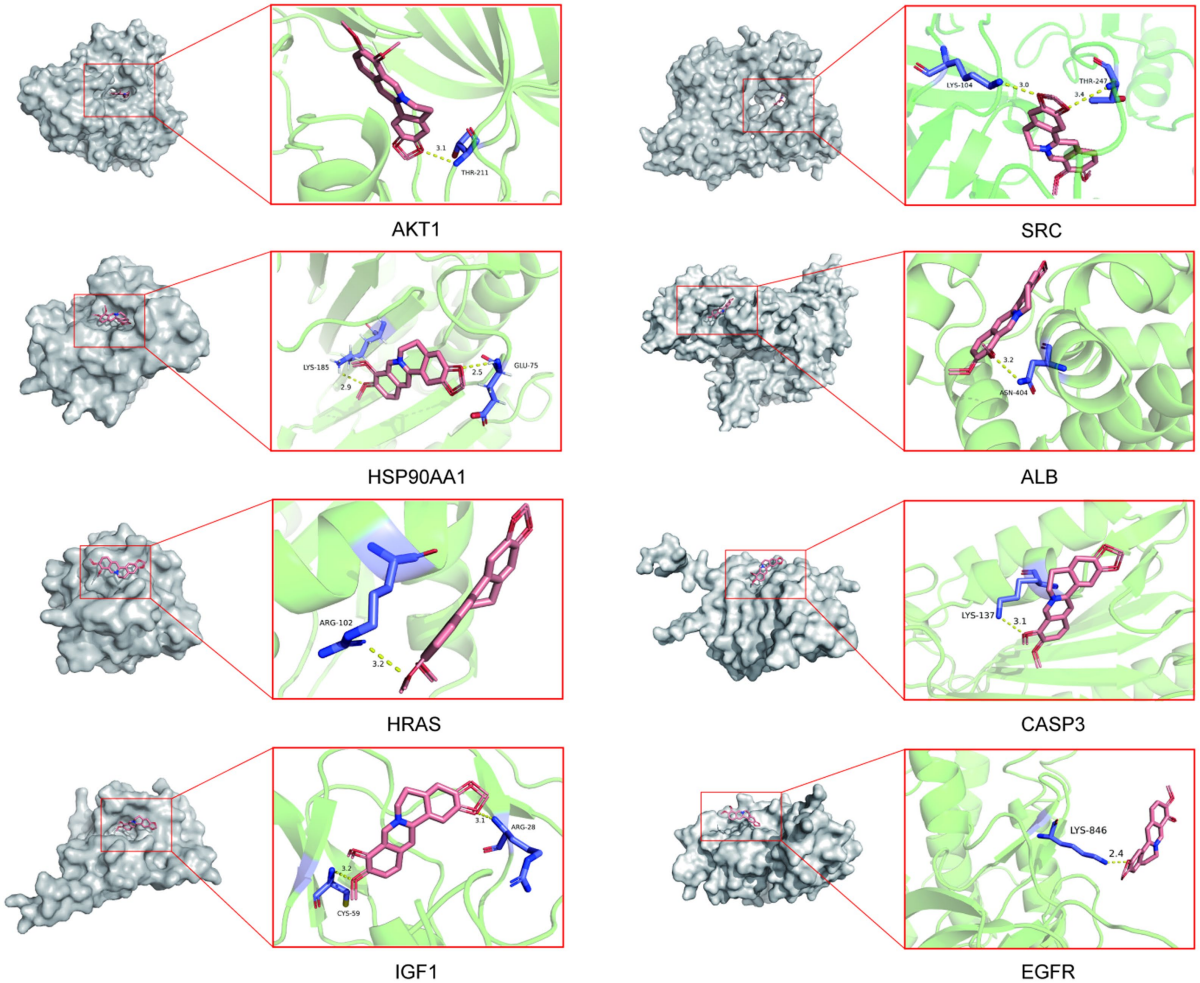
Molecular docking results of core protein targets with BBR. The dotted line represents hydrogen bond interaction.

To validate the core targets of BBR in AD, semi-flexible molecular docking was performed using Auto Dock Vina software. A lower affinity score indicates a stronger binding force. The crystal structures of the target proteins are listed below: AKT1 (PDB: 4ejn), HSP90AA1 (PDB: 3t0h), SRC (PDB: 1fmk), HRAS (PDB: 2ce2), IGF1 (PDB: 1wqj), ALB (PDB: 6hn1), CASP3 (PDB: 2dko), EGFR (PDB: 8a27). After molecular docking and interaction analysis, BBR was found to bind in a stable conformation to its targets as indicated by the low affinity scores: −10.2 kcal/mol (AKT1), −6.5 kcal/mol (HSP90AA1), −9.6 kcal/mol (SRC), −5.2 kcal/mol (HRAS), −7.8 kcal/mol (IGF1), −8.4 kcal/mol (ALB), −6.6 kcal/mol (CASP3) and − 9.5 kcal/mol (EGFR). All RMSD values were less than 2A, suggesting the molecular docking results are reliable (Table 2). Docking of core targets and BBR is shown in Figure 7.

**Table 2:** Molecular docking results of core targets and BBR.

Target name	PDB	Affinity (kcal/mol)	RMSD (A)
AKT1	4ejn	−10.2	0.001
HSP90AA1	3t0h	−6.5	0.000
SRC	1fmk	−9.6	0.000
HRAS	2ce2	−5.2	0.001
IGF1	1wqj	−7.8	0.000
ALB	6hn1	−8.4	0.000
CASP3	2dko	−6.6	0.001
EGFR	8a27	−9.5	0.000

PDB, Protein Data Bank; RMSD, Root Mean Square Deviation.

### BBR acts through the AKT pathway

**Figure 8:**
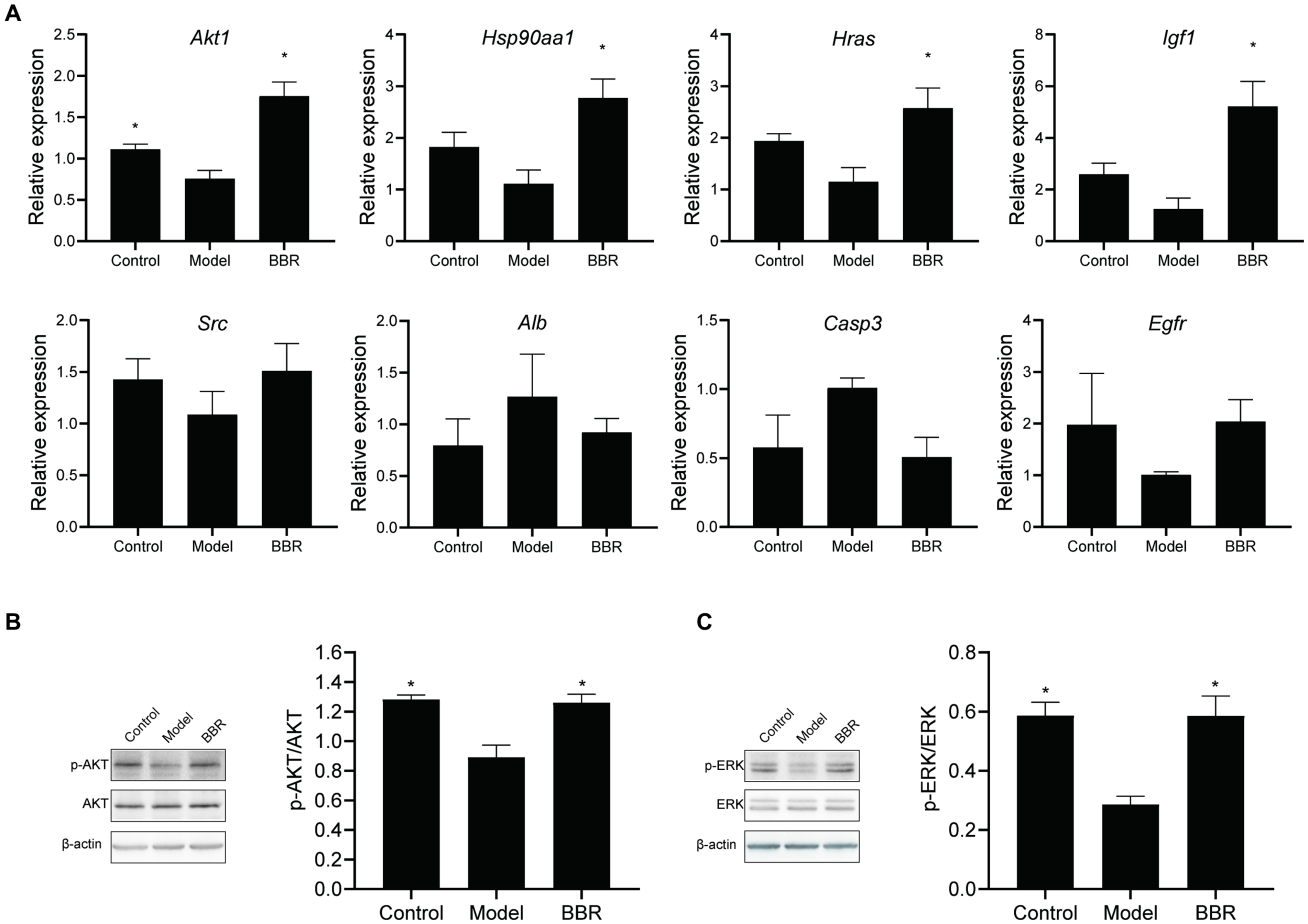
BBR acts through the AKT pathway. **(A)** Relative expression changes of the core targets; **(B)** BBR increased AKT phosphorylation; **(C)** BBR increased ERK phosphorylation. Data were analyzed by *t* test between two groups, presented as mean ± SEM, *n* = 4, *p < 0.05 compared to the model group.

Relative expression changes of the core targets were detected by RT-qPCR (Figure 8A). The level of *Akt1* mRNA was significantly reduced in AD mice (*t* = 2.96, *p* = 0.025). A similar trend was observed for *Hsp90aa1*, *Hras*, *Igf1*, and *Src* mRNA levels, but none of them was statistically significant (*p* > 0.05). Among the core targets, *Akt1* (*t* = −5.01, *p* = 0.002), *Hsp90aa1* (*t* = −3.66, *p* = 0.011), *Hras* (*t* = −2.99, *p* = 0.024) and *Igf1* (*t* = 3.75, *p* = 0.019) mRNA levels were significantly increased after BBR treatment, while *Src*, *Egfr* mRNA levels showed a similar trend but lacked statistical significance (*p* > 0.05). *Alb*, *Casp3* mRNA levels showed the opposite trend. These results suggest that *Akt1*, *Hsp90aa1*, *Hras*, *Igf1* could play important roles in BBR treatment of AD.

Then, we explored whether BBR affects protein phosphorylation of AKT as well as ERK by western blotting at the protein assay level. As shown in Figures 8B,C, AKT (*t* = 4.489, *p* = 0.004) and ERK (*t* = 5.645, *p* = 0.001) phosphorylation decreased in the model group, and BBR significantly increased AKT (*t* = −3.721, *p* = 0.010) and ERK (*t’* = −4.126, *p* = 0.014) phosphorylation levels.

## Discussion

The molecular characteristics and physicochemical properties of BBR indicate good druggability (Kumar et al., 2015). The properties of BBR suggest that it may be able to cross the blood–brain barrier (BBB) thus facilitating direct binding to potential targets within the central nervous system (Peng et al., 2004; Jiang et al., 2015). After intravenous administration, BBR is quickly removed from the plasma (t_1/2β_ = 1.13 h) and sharply increased in the hippocampus (t_1/2α_ = 0.215 h) with a delayed clearance rate (t_1/2β_ = 12.0 h), indicating that it has the potential to act instantly through BBB, and can be stored in the hippocampus, affecting memory and recognition performance (Wang et al., 2005).

Several *in vitro* and *in vivo* experiments have revealed that BBR elicits neuroprotective effects in AD models as indicated by the following lines of data: ① BBR reduces Aβ levels by modulating APP processing and ameliorates Aβ pathology *by* inhibiting the mTOR/p70S6K signaling pathway (Asai et al., 2007; Durairajan et al., 2012; Panahi et al., 2013; Wang et al., 2021); ② it inhibits tau hyperphosphorylation at Thr205 and Thr231 in the hippocampus *via* the GSK3β/PGC-1α (Yang and Wang, 2022) or NF-κB signaling pathways (He et al., 2017); ③ it reduces the production of pro-inflammatory cytokines in activated microglial cells and modulates mitochondrial bioenergetics (He et al., 2017; Wong et al., 2021); ④ it reduces lactate dehydrogenase release and reactive oxygen species (ROS) generation (Chen et al., 2015), as well as the relative mRNA expression of endoplasmic reticulum (ER) stress related pathway genes (Xuan et al., 2020); ⑤ it rescues synaptic damage by activating LKB1/AMPK signaling in AD mice (Cai et al., 2019); ⑥ it promotes the formation of brain microvessels by enhancing CD31, VEGF, N-cadherin, Ang-1 and inhibits neuronal apoptosis (Ye et al., 2021). Consistent with a previous study (Ye et al., 2021), our work shows that BBR improves memory and recognition performance in AD mice. Furthermore, we show that BBR also ameliorates neuronal damage in the hippocampus. These results suggest that BBR plays a potential therapeutic role in AD by protecting hippocampal neurons. However, the specific targets and mechanisms involved in this protective effect are currently poorly understood and more basic and clinical studies will be needed to confirm these results. In addition, BBR, together with epiberberine, coptisine, palmatine, and gatrorrhizine are the main active constituents of *Coptis chinensis* Franch (Huanglian), participating in the prevention and treatment of AD (Meng et al., 2018; Wang et al., 2020). Among them, BBR is the component with the highest content. Due to the similarity of physicochemical properties of these substances, the purification and separation process of BBR by conventional methods such as acid-water and ethanol inevitably incorporates other impurities, and it is difficult to achieve 100% purity (Sun et al., 2014). Although most of the commercially purchased BBR products can reach a purity of 98% or more, there are still less than 2% impurities that may have undeniable influence on the study results. Thus, HPLC purification to remove contaminants would need to be performed to ensure activities observe are not from these impurities in a more in-depth study in the future.

Network pharmacology is considered the next paradigm in drug discovery because it allows to analyze the “herb-compound-protein/gene-disease” interaction network from a systems biology perspective. Therefore, we investigated the targets of the action of BBR in AD using network pharmacology. 117 BBR-associated targets of AD were identified, of which 43 targets were selected for downstream functional analysis by protein–protein interaction analysis using MCODE and cytoHubba. The PI3K-AKT signaling pathway and MAPK signaling pathway are members of the most significant terms identified. *ALB, EGFR, CASP3* and five targets that are members of the PI3K-AKT pathway, including *AKT1, HSP90AA1, SRC, HRAS*, *IGF1* were extracted as core targets of BBR in AD.

AKT, which functions downstream of PI3K, is the core component of the PI3K-AKT pathway. Many cytokines or growth factors induce phosphorylation of tyrosine residues by binding to the RTK membrane receptor. The regulatory subunit of PI3K, P85, binds to phosphorylated tyrosine residues through its SH2 domain, which in turn recruits the catalytic subunit P110 to form the active PI3K enzyme. Activated PI3K further acts on PDK1, PIP3, to promote AKT phosphorylation (Thorpe et al., 2015; Long et al., 2021). AKT, also known as protein kinase B (PKB), is a serine/threonine kinase participating in the PI3K-Akt pathway. AKT1/PKBα is encoded by *AKT1*, which is composed of three isoforms that contain a conserved plekstrin homology domain, a central fragment, and a regulatory domain (Hanada et al., 2004). Thr308 and Ser473 are essential phosphorylation sites for AKT activation (Wei et al., 2019). HSP90AA1, SRC, HRAS, and IGF1 are members of the PI3K-AKT pathway. The heat shock protein (Hsp) 90 encoded by HSP90AA is a molecular chaperone involved in protein folding that regulates the activity of AKT (Jhaveri and Modi, 2015). In the brain, Hsp90 is involved in synaptic plasticity (Chen et al., 2014) as well as in the targeting of tau for proteosomal degradation (Dickey et al., 2007), regulate Aβ processing (Blair et al., 2014). Hsp90 usually forms a protein complex with other co-chaperones Hsp70, Hsp60, Hsp23, acting as the core of the complex to promote the activation or stabilize the conformation of the target protein. Inhibitors of Hsp90 bind to the regulatory site of Hsp90 and act on it. There are two possible pathways: one occurs by inducing a cytoprotective heat shock response, and the other acts by directing the degradation of pathogenic proteins. In AD, Hsp90 expression is downregulated, and after administration of Hsp90 inhibitor, Aβ-induced neurotoxicity could be prevented by increasing the level of Hsp70 and Hsp90 (Ansar et al., 2007; Gezen-Ak et al., 2013; Ou et al., 2014). The SRC protein-tyrosine kinase family mainly consists of 11 members, including SRC, FYN, and YES, three closely related group I enzymes (Brown and Cooper, 1996). Members of the SRC kinase family are controlled by cytokine receptors, G-protein coupled receptors, etc., participating in pathways that regulate survival, proliferation, and regulation of gene expression through AKT or MAPKs (Thomas and Brugge, 1997; Roskoski, 2015). Recently, FYN was shown to have several functions in the central nervous system (CNS), and FYN dysfunction has been implicated in the pathological processes leading to AD (Nygaard, 2018). Rho GTPases, including HRAS, have significant therapeutic potential in the treatment of neurodegenerative diseases, as they have been implicated in nearly all stages of brain development (Zamboni et al., 2018; Schmidt et al., 2022). IGF1 may have a therapeutic effect in neurodegenerative disorders by enhancing hippocampal neurogenesis (Morel et al., 2017) and promoting neuronal survival through the PI3K / AKT and MAPK pathways (Vincent et al., 2004; Bronzi et al., 2010). Extracellular-signalregulated protein kinase (ERK), as one of the MAPK signaling subfamilies, is usually downstream of the AKT pathway. Phosphorylation of ERK1/2 protein activates the ERK pathway and plays an important role in regulating cell growth and differentiation (Kumar et al., 2023).

The *ALB* gene encodes the most abundant protein in the blood, which is albumin. Higher levels of albumin in the cerebrospinal fluid (CSF) of AD patients respond to damage to the BBB (Lin et al., 2021). It can differentiate AD patients from controls together with hub genes including *JUN*, *SLC2A1*, *TFRC*, and *NFE2L2* (Wang et al., 2022). *EGFR* is a potential therapeutic target for AD (Tsuji et al., 2021; Zhang et al., 2022).The protein encoded by *CASP3* gene is a cysteine-aspartic acid protease that plays an important role in the execution-phase of cell apoptosis in AD, and the PI3K/AKT signaling pathway is the most important intracellular factor involved in regulation of cellular apoptosis (Khezri et al., 2023). In addition, it can be activated upstream by the presence of extracellular factors and then act downstream on tau protein (Avila, 2010). However, interactions obtained using STRING for PPI analysis are not specific for brain, which may interfere with the specificity of the targets and the subsequent validation results.

To validate identified core targets, we performed molecular docking analysis using AutoDock Vina (Trott and Olson, 2010), a computational method for predicting bound conformations and binding affinity. The results showed strong binding between BBR and the identified core targets. This suggests to us that BBR may play a role by altering the function of these proteins through compound binding. Furthermore, we determined the relative expression changes of the main targets in AD mice by RT-qPCR, and found that *Akt1, Hsp90aa1, Hras*, and *Igf1* mRNA levels were significantly altered after BBR treatment. In addition, our results confirm that BBR significantly increased AKT and ERK phosphorylation levels. Phosphorylated AKT activates a range of downstream pathways, including the P53 pathway, regulating protein translation, cell cycle, apoptosis. Activation of PI3K/AKT signaling protects neurons from Aβ-induced neurotoxicity (Do et al., 2014), inhibits the formation of pathogenic neurogenic fiber tangles (NFT) (Kitagishi et al., 2014), and regulates synaptic plasticity (Spinelli et al., 2019), playing an important regulatory role in AD. Phosphorylation of ERK1/2 protein activates the ERK pathway, and accumulation of intra-neuronal (Aβ1–42) causes the significant reduction in phosphorylation of ERK1/2 protein, affecting neuronal viability (Cruz et al., 2018; Kumar et al., 2023). Taken together, these results suggest that the ERK and AKT signaling pathways are crucial pathways to mediate the therapeutic effects of BBR in AD mice.

BBR can have multiple effects in the treatment of AD, and this is important because of the complex pathological changes and symptoms at different stages of AD. Given the low water solubility and limited gastrointestinal absorption of BBR, nanotechnological modifications of BBR and its use in combination with other drugs may be useful to improve both bioavailability and therapeutic efficacy (Singh A. et al., 2021; Singh et al., 2021b; Behl et al., 2022). More in-depth studies are needed in the future.

## Conclusion

*AKT1*, *HSP90AA1*, *SRC*, *HRAS*, *IGF1*, and *ALB*, *EGFR*, *CASP3* were core targets of BBR in the treatment of AD. BBR can exert a neuroprotective effect by modulating the ERK and AKT signaling pathways.

## Data availability statement

The original contributions presented in the study are included in the article/Supplementary material, further inquiries can be directed to the corresponding authors.

## Ethics statement

The animal study was reviewed and approved by the Ethics Committee of Xiyuan Hospital of China Academy of Chinese Medical Sciences.

## Author contributions

WW and J-xY performed experiments and data analysis and wrote the manuscript. T-tZ, J-yW, ZZ, and Y-mL assisted in the experiments. YC and HL assisted with ideas and modification of the manuscript. All authors reviewed and approved the manuscript.

## Funding

This study was funded National Key R&D Program of China (2022YFC3501400), and the Fundamental Research Funds for the Central public welfare research institutes (ZZ15-YQ-013 and ZZ15-XY-PT-02), and science and technology innovation project of Chinese Academy of traditional Chinese Medicine (CI2021A01401 and CI2021A04618).

## Conflict of interest

The authors declare that the research was conducted in the absence of any commercial or financial relationships that could be construed as a potential conflict of interest.

## Publisher’s note

All claims expressed in this article are solely those of the authors and do not necessarily represent those of their affiliated organizations, or those of the publisher, the editors and the reviewers. Any product that may be evaluated in this article, or claim that may be made by its manufacturer, is not guaranteed or endorsed by the publisher.

## Supplementary material

The Supplementary material for this article can be found online at: https://www.frontiersin.org/articles/10.3389/fnins.2023.1059496/full#supplementary-material

## Footnotes

^1^ https://tcmsp-e.com/tcmsp.php

^2^ https://pubchem.ncbi.nlm.nih.gov/compound/2353

^3^ http://lilab-ecust.cn/pharmmapper/

^4^ http://ctdbase.org/about/

^5^ https://www.disgenet.org/

^6^ https://www.genecards.org/

^7^ https://string-db.org/

^8^ https://www.rcsb.org/

^9^ https://pymol.org/2/

^10^ http://vina.scripps.edu/
