# Supplementary material for: Network pharmacology reveals that Berberine may function against Alzheimer’s disease via the AKT signaling pathway
Source: Front Neurosci. 2023 May 4;17:1059496. doi: 10.3389/fnins.2023.1059496 (PMC10192713; doi:10.3389/fnins.2023.1059496)
Supplement: Supplementary file 5 [file Data_Sheet_1.docx]

Table 1. Comparison of open field test results

| Group | Control (n=9) | Model (n=9) | BBR (n=7) |
| --- | --- | --- | --- |
| Total distance (cm) | 827.21±184.36^*^ | 2180.19±196.21 | 1142.32±208.98^*^ |
| Total move time (s) | 49.53±10.26^*^ | 132.17±15.69 | 71.56±9.37^*^ |
| Speed (cm/s) | 2.76±0.61^*^ | 7.27±0.65 | 3.81±0.70^*^ |
| Distance in center (cm) | 50.95±12.63 | 98.00±15.74 | 41.65±16.18^*^ |
| Time in center (s) | 2.61±0.68 | 4.25±0.75 | 1.82±0.72^*^ |

Notes: Data are presented as mean with SEM using *t* test, with **P*<0.05 compared to the model group.

Table 2. Comparison of novel object recognition test results

| Group | Control (n=9) | Model (n=9) | BBR (n=7) |
| --- | --- | --- | --- |
| PI (preference index) % | 2.06±0.59 | 1.80±0.54 | 1.68±0.46 |
| RI (recognition index) % | 0.70±0.05^*^ | 0.39±0.06 | 0.57±0.04^*^ |

Notes: Data are presented as mean with SEM using *t* test, with **P*<0.05 compared to the model group.

Table 3 Latency results of experimental positioning navigation for each group of mice

| Groups | Positioning navigation experiment | | | | |
| --- | --- | --- | --- | --- | --- |
|  | DAY1 | DAY2 | DAY3 | DAY4 | DAY5 |
| Control | 47.27±3.42^◆^ | 45.05±5.18^◆^ | 21.41±6.59^◆#^ | 36.15±7.33^◆^ | 25.19±6.02^◆#^ |
| Model | 90.00±3.42 | 82.19±5.18 | 68.79±6.59^#^ | 66.80±7.33^#^ | 59.91±6.02^#^ |
| BBR | 72.55±3.88^◆^ | 60.45±5.86^◆#^ | 55.76±7.47 | 49.83±8.32^#^ | 32.74±6.82^◆#^ |

Note: Latency data were presented as mean with SEM using ANOVA with repeated measurement data, with ^◆^*P*<0.05 for latency data between groups on the same day compared with the model group; and ^#^*P*<0.05 for latency data within groups compared with the first day.

Table 4 Results of spatial exploration experiments for each group of mice

| Groups | Space exploratory experiments | | |
| --- | --- | --- | --- |
|  | Platform crossover number | Distance in target quadrant (%) | Time in target quadrant (%) |
| Control | 5.67±1.47* | 0.38±0.05 | 0.37±0.05 |
| Model | 2.22±0.57 | 0.30±0.02 | 0.32±0.02 |
| BBR | 4.00±0.49* | 0.27±0.03 | 0.29±0.03 |

Note: Data are presented as mean with SEM using *t* test, with **P*<0.05 compared to the model group.
